# Intermittent montelukast in children aged 10 months to 5 years with wheeze (WAIT trial): a multicentre, randomised, placebo-controlled trial

**DOI:** 10.1016/S2213-2600(14)70186-9

**Published:** 2014-10

**Authors:** Chinedu Nwokoro, Hitesh Pandya, Stephen Turner, Sandra Eldridge, Christopher J Griffiths, Tom Vulliamy, David Price, Marek Sanak, John W Holloway, Rossa Brugha, Lee Koh, Iain Dickson, Clare Rutterford, Jonathan Grigg

**Affiliations:** a Asthma UK Centre for Applied Research, Queen Mary University of London, London, UK; b Centre for Primary Care and Public Health, Blizard Institute, Queen Mary University of London, London, UK; c Department of Infection, Immunity and Inflammation, University of Leicester, Leicester, UK; d Institute of Applied Health Sciences, University of Aberdeen, Aberdeen, UK; e Department of Medicine, Jagiellonian University Medical School, Krakow, Poland; f Human Development and Health, University of Southampton, Southampton General Hospital, UK

## Abstract

**Background:**

The effectiveness of intermittent montelukast for wheeze in young children is unclear. We aimed to assess whether intermittent montelukast is better than placebo for treatment of wheeze in this age group. Because copy numbers of the Sp1-binding motif in the arachidonate 5-lipoxygenase (*ALOX5*) gene promoter (either 5/5, 5/x, or x/x, where x does not equal 5) modifies response to montelukast in adults, we stratified by this genotype.

**Methods:**

We did this multicentre, parallel-group, randomised, placebo-controlled trial between Oct 1, 2010, and Dec 20, 2013, at 21 primary care sites and 41 secondary care sites in England and Scotland. Children aged 10 months to 5 years with two or more wheeze episodes were allocated to either a 5/5 or 5/x+x/x *ALOX5* promoter genotype stratum, then randomly assigned (1:1) via a permuted block schedule (size ten), to receive intermittent montelukast or placebo given by parents at each wheeze episode over a 12 month period. Clinical investigators and parents were masked to treatment group and genotype strata. The primary outcome was number of unscheduled medical attendances for wheezing episodes. Analysis was by intention to treat. This trial is registered with ClinicalTrials.gov, number NCT01142505.

**Findings:**

We randomly assigned 1358 children to receive montelukast (n=669) or placebo (n=677). Consent was withdrawn for 12 (1%) children. Primary outcome data were available for 1308 (96%) children. There was no difference in unscheduled medical attendances for wheezing episodes between children in the montelukast and placebo groups (mean 2·0 [SD 2·6] *vs* 2·3 [2·7]; incidence rate ratio [IRR] 0·88, 95% CI: 0·77–1·01; p=0·06). Compared with placebo, unscheduled medical attendances for wheezing episodes were reduced in children given montelukast in the 5/5 stratum (2·0 [2·7] *vs* 2·4 [3·0]; IRR 0·80, 95% CI 0·68–0·95; p=0·01), but not in those in the 5/x+x/x stratum (2·0 [2·5] *vs* 2·0 [2·3]; 1·03, 0·83–1·29; p=0·79, p_interaction_=0·08). We recorded one serious adverse event, which was a skin reaction in a child allocated to placebo.

**Interpretation:**

Our findings show no clear benefit of intermittent montelukast in young children with wheeze. However, the 5/5 *ALOX5* promoter genotype might identify a montelukast-responsive subgroup.

**Funding:**

Medical Research Council (UK) and National Institute for Health Research.

## Introduction

Wheeze in children aged 10 months to 5 years is characterised by recurrent episodes that are frequently triggered by viral colds.^1^ Episodes of wheeze in young children might be clinically severe and can result in parents seeking medical attention.^2^ Indeed, an audit of UK paediatric hospital admissions for acute wheeze from 1998 to 2005 showed that most admissions were of children younger than 5 years.^3^ Because wheeze in young children is characterised by long asymptomatic periods interspersed with short intense episodes,^1^ intermittent treatment strategies have been assessed. We previously reported that a short course of oral corticosteroids initiated by parents at the onset of a wheeze episode is not effective for reducing the severity of wheeze in children aged 1–5 years.^4^ By contrast, intermittent high-dose inhaled corticosteroids reduce the risk of clinically severe wheeze episodes by 30% in that age group.^5^ However, this strategy is associated with clinically relevant growth suppression.^5^ Because montelukast (a cysteinyl leukotriene receptor blocker) does not suppress growth,^6^ the effectiveness of intermittent montelukast for wheeze in young children is of clinical interest.

To date, trials of intermittent montelukast have reported conflicting results: findings from a subgroup analysis in Robertson and colleagues' trial^7^ of children aged 2–14 years showed that intermittent montelukast given over 12 months reduced unscheduled use of acute health-care resources by 38%; Bacharier and colleagues^8^ reported that intermittent montelukast therapy over 12 months does not decrease wheeze severity in young children or need for oral corticosteroid therapy; and Valovirta and colleagues^9^ reported no beneficial effect of a 12 month course of intermittent montelukast on wheeze attacks in young children. Reasons for these inconsistent results could be the substantial heterogeneity in treatment effect in young children with wheeze,^10^ and that the response to montelukast is limited to a subgroup of children.

Studies of adults with asthma suggest that heterogeneity in response to montelukast is partly determined by a polymorphism in the arachidonate 5-lipoxygenase (*ALOX5*) gene promoter. The *ALOX5* gene encodes 5-lipoxygenase—the rate-limiting enzyme in the cysteinyl leukotriene biosynthetic pathway.^11^, ^12^ This polymorphism results from variable numbers of copies of the Sp1 binding motif GGGCGG, whereby five Sp1 repeats are the major allele.^13^ Thus individuals are classified as either 5/5, or 5/x (in which x is not equal to 5), or x/x.^14^ To date, the *ALOX5* promoter genotype grouping that best defines montelukast responsiveness in adults is unclear. For example, Telleria and colleagues^15^ reported increased montelukast responsiveness in adults with the 5/5 and the 5/x genotype (compared with x/x), whereas Lima and colleagues^14^ reported that both the 5/x and x/x genotypes were responsive to montelukast.

We did the Wheeze And Intermittent Treatment (WAIT) trial to assess the efficacy of intermittent montelukast for wheeze in young children at increased risk of clinically severe episodes of wheeze.

## Methods

### Study design and participants

We did this multicentre, parallel-group, randomised, placebo-controlled trial between Oct 1, 2010, and Dec 20, 2013, at 21 primary care sites and 41 secondary care sites in England and Scotland. Eligible children were aged between 10 months and 5 years and had had two or more previous episodes of wheeze, at least one of which was physician-confirmed, and at least one of which had happened in the preceding 3 months. We excluded children if they had a pre-existing respiratory vulnerability such as cystic fibrosis, sickle-cell disease, severe developmental delay with feeding difficulty, history of neonatal chronic lung disease, or structural airways disease. Children were also excluded if they had been enrolled in a therapeutic trial in the previous 3 months or were taking continuous oral montelukast at the time of enrolment. To represent the overall population of young children with wheeze, and in line with the population in our previous trials,^4^, ^16^ we did not exclude children receiving continuous inhaled corticosteroid therapy. The study was approved by the UK National Health Service Multicenter Research Ethics Committee (reference number 09/H1102/110), by local institutional review boards, and by the UK Medicines and Healthcare Products Regulatory Agency (21313/0024/01-0001); the UK Medicines for Children Research Network supported the study. An independent data and safety monitoring committee not involved with patient enrolment reviewed adverse events. Written informed consent was obtained from the parent or guardian of each child enrolled in the study.

### Randomisation and masking

Participants were allocated to either a 5/5 or 5/x+x/x *ALOX5* promoter genotype stratum, then randomly assigned (1:1), via a permuted block schedule (size ten) developed by the manufacturer (Novalabs, Leicester, UK), to receive montelukast or placebo (appendix). Clinical investigators and parents were masked to treatment group and genotype strata. Placebo and montelukast were packaged as identical granules in identical sachets labelled with participant number only. Emergency code break was allowed in cases of a suspected severe adverse reaction when knowledge of patient allocation could have affected clinical management of a study participant, in the case of a suspected unexpected severe adverse reaction, and in any other circumstance in which the principal investigator considered that an emergency code break was indicated.

### Procedures

At enrolment, parents completed a structured questionnaire administered by research study personnel, which asked about previous wheeze, present treatment, and risk factors (appendix). Saliva from each child was collected with the Oragene OG-250 collection kit in combination with the CS-1 saliva collection kit for young children (both manufactured by DNA Genotek, Ottawa, ON, Canada) and transferred to Queen Mary University of London (London, UK) for analysis. The simple sequence-length polymorphism in the promoter region of *ALOX5* (rs59439148) was genotyped as described previously.^17^ Alleles were classified according to the number of simple repeats (appendix), and children were identified as belonging to either 5/5 or 5/x+x/x strata.

Parents were advised to commence the trial drug at the onset of each viral cold or wheezing episode over the 12-month study period. Parents continued all other drugs prescribed by their managing clinician (including bronchodilators and inhaled corticosteroids), and completed a diary of symptoms, medicine use, adverse events, and medical attendance for each day the trial drug was given (appendix). Investigators asked parents by telephone survey about usage of trial drug, use of oral corticosteroid rescue therapy, and medical attendances at two-monthly intervals during the 12-month study period (appendix). Parents who could not be contacted received a maximum of two letters offering continued involvement in the study. When parents could not be contacted for two successive phone calls, parent and child were regarded as withdrawn from the study. Medical attendances for wheeze were independently verified by study investigators by contact with the managing clinician.

Urine was obtained from asymptomatic children at baseline. Urine was transported on ice, and stored at −80°C within 1 h of collection. We analysed urinary leukotriene E_4_—the final urinary metabolite of cysteinyl leukotriene production—by high-performance liquid chromatography–tandem mass spectrometry (ABI SCIEX 4000 QTRAP, Framingham, MA, USA), as previously described (appendix).^18^ Concentrations were expressed in proportion to urinary creatinine. We excluded samples with a urinary creatinine concentration of less than 0·1 mg/mL because correction is inaccurate in very dilute samples.

We monitored children for adverse events with a diary card report and telephone follow-up. Hospital admission for exacerbation of wheeze, acute lower-respiratory-tract infection, acute febrile illness, febrile convulsion, gastroenteritis, and exacerbation of eczema were not classed as serious adverse events in the trial protocol.

### Outcomes

Our primary outcome was the number of unscheduled medical attendances for wheezing episodes. Such attendances were defined as those to a family doctor, an asthma nurse or similarly trained health-care professional, an accident and emergency department, hospital via accident and emergency (hospital admission), or any combination of these. Secondary outcomes were duration of hospital admission, number of wheeze episodes, duration of wheeze episodes, number of courses of oral steroids per year, proportion of children receiving oral corticosteroids, use of trial drug, time to first unscheduled medical attendance, and time to first unscheduled attendance by site of medical attendance. We did a prespecified subgroup analysis that assessed unscheduled medical attendances for wheeze episodes by *ALOX5* promoter genotype strata (5/5 and 5/x+x/x). Other prespecified subgroups for analysis were multitrigger and episodic wheeze at baseline, use of either continuous inhaled corticosteroids or no inhaled corticosteroids at baseline, and the alternative genotype grouping of 5/5+5/x and x/x.

### Statistical analysis

The trial was powered to detect a difference in the number of unscheduled medical attendances for wheeze episodes between participants in the intervention and control groups, and to detect differential responsiveness to montelukast in the 5/5 stratum compared with the 5/x+x/x stratum, with the assumption that montelukast leads to a 60% reduction in attendances in the 5/x+x/x stratum, and a 20% reduction in the 5/5 stratum. With use of data from the UK General Practitioner Research Database, with courses of oral steroids as a proxy for unscheduled medical attendances for wheeze episodes, we estimated a mean of 0·76 [SD 1·22] such attendances per year. Because data follow an overdispersed Poisson distribution, we used Markov chain Monte Carlo simulation in WinBUGs (version 1.4) to estimate required sample sizes. 1050 children were needed to detect a 33% drop in unscheduled medical attendances for wheeze episodes, with a power of 90% at a significance level of 5%, with a 6% loss to follow up. A 33% drop in attendances equates to an attack rate of 0·51 for the treatment group. The clinical significance of these changes is that roughly four children would need to be treated to prevent one unscheduled medical attendance. Because a sample size of 1200 provides just more than 80% power at the 5% significance level to detect an interaction between treatment and *ALOX5* genotype, 1300 children needed to be recruited, assuming a 6% dropout. Interim safety analyses were done at 6-monthly intervals. Efficacy analyses were done at the end of the trial.

For each child, we analysed unscheduled medical attendances for wheeze episodes and episodes of viral cold with a Poisson regression model. For each episode of wheeze and viral cold, duration of hospital admission, and number of symptom days were also analysed with Poisson regression models. We included follow-up time for each child as an exposure variable and a random effect fitted for each child to account for overdispersion or when episode was the unit of analysis. Follow-up time was based on time from randomisation until either the 12 month end of trial date or date of last phone call.

For unscheduled medical attendances for wheeze episodes, we assessed the differential effect of treatment in predefined subgroups by inclusion of an interaction term. Proportions of patients who had any unscheduled medical attendance, or those receiving oral corticosteroid rescue therapy, were analysed with logistic regression. We analysed time to first unscheduled medical attendance with Cox regression models. All models were fitted on the available case population with modified intention-to-treat principles and included fixed effects for stratification factor and treatment. We did a per-protocol analysis that excluded any children randomised not according to schedule and that corrected for those randomised under the incorrect stratum. Parents who withdrew their children from the study and provided permission to use their data were included in the analysis to the point of withdrawal. Parents who withdrew their children and did not provide permission for their data to be used were excluded from the analysis. Because we anticipated few missing data, no imputation of missing data was done. All analyses were two-sided with a 5% significance level. Results are presented as incidence rate ratios (IRRs), odds ratios (ORs), or hazard ratios (HRs) as appropriate, with corresponding 95% CIs. To assess the effect of *ALOX5* genotype on urinary leukotriene E_4_, data were first log_10_ transformed to normalise distribution. Groups were compared with either ANOVA and Dunnett's multiple comparisons test, or with *t* test using GraphPad Prism version 6.00 for Windows (GraphPad Software, La Jolla, CA, USA). Analyses were done with STATA Statistical Software: release 12.1. This trial is registered with ClinicalTrials.gov, number NCT01142505.

### Role of funding source

The sponsor of the study had no role in study design, data collection, data analysis, data interpretation, or writing of the report. All authors had full access to all raw data and the corresponding author had full access to all the data in the study and had final responsibility for the decision to submit for publication.

## Results

**Figure 1:**
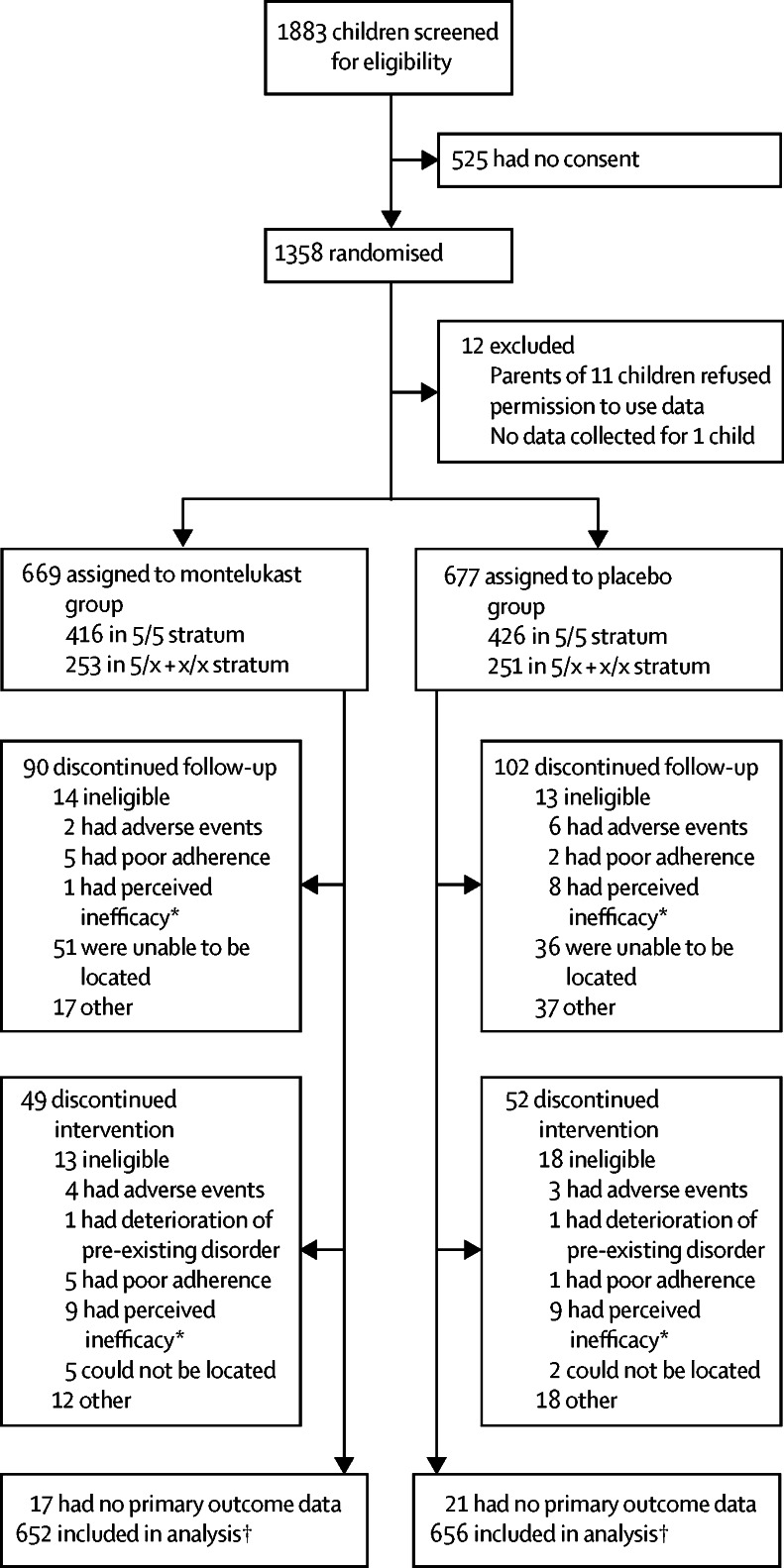
Trial profile *Perceived inefficacy is on the side of patient. †Data for the primary outcome were obtained from children whose parents responded to at least one follow-up phone call.

Figure 1 shows the trial profile. Parents of 1366 children provided consent to enter the study, of whom eight withdrew children before randomisation. The remaining 1358 children were randomly assigned to receive montelukast (n=669) or placebo (n=677; figure 1). Data for the primary outcome were obtained from 1308 (96%) children whose parents responded to at least one follow-up phone call (figure 1). Baseline demographic characteristics were similar between treatment groups. The per-protocol analysis included 1297 children. 11 children were excluded who had been incorrectly randomised and the strata was corrected for two children who were randomised with incorrect strata. There were no major differences in baseline variables between children in the placebo and montelukast groups or between the two genetic strata (table 1). The dominant allele was five repeats (table 1), and consistent with previous reports,^14^, ^19^ black children had a greater frequency of x alleles (75% *vs* 31% in white children; appendix).

**Table 1:** Baseline characteristics

		**Montelukast group (n=669)**			**Placebo group (n=677)**		
		5/5	5/x+x/x	Total	5/5	5/x+x/x	Total
n (%)		416 (62%)	253 (38%)	669 (100%)	426 (63%)	251 (37%)	677 (100%)
Height (cm)		90·0 (10·3)	89·8 (10·5)	89·9 (10·4)	89·9 (10·5)	91·8 (11·7)	90·6 (11·0)
Weight (kg)		14·0 (3·0)	13·9 (3·7)	14·0 (3·3)	14·0 (3·3)	14·6 (3·8)	14·2 (3·5)
Age (years)		2·6 (1·1)	2·5 (1·1)	2·6 (1·1)	2·6 (1·1)	2·8 (1·2)	2·7 (1·1)
Male sex		262 (63%)	164 (65%)	426 (64%)	276 (65%)	161 (64%)	437 (65%)
Ethnic origin							
	White	335 (81%)	179 (71%)	514 (77%)	338 (79%)	174 (69%)	512 (76%)
	Black	5 (1%)	14 (6%)	19 (3%)	4 (1%)	14 (6%)	18 (3%)
	Asian	55 (13%)	37 (15%)	92 (14%)	58 (14%)	46 (18%)	104 (15%)
	Other	21 (5%)	23 (9%)	44 (7%)	26 (6%)	17 (7%)	43 (6%)
Preterm birth (<37 weeks)		58 (14%)	40 (16%)	98 (14%)	56 (13%)	42 (17%)	98 (15%)
Birthweight (<2500g)		51 (12%)	28 (11%)	79 (12%)	42 (10%)	28 (11%)	70 (10%)
Food allergy		64 (15%)	44 (18%)	108 (16%)	64 (15%)	47 (19%)	111 (17%)
Drug allergy		26 (6%)	12 (5%)	38 (6%)	23 (6%)	19 (8%)	42 (6%)
Itchy rash (>6 months, ever)*		98 (23%)	64 (25%)	162 (24%)	104 (25%)	60 (24%)	164 (25%)
Eczema (ever)†		207 (49%)	121 (48%)	328 (48%)	215 (52%)	134 (53%)	349 (52%)
History of asthma in mother		156 (37%)	95 (38%)	251 (37%)	141 (34%)	89 (35%)	230 (34%)
History of asthma in father		126 (30%)	73 (29%)	199 (29%)	126 (30%)	81 (32%)	207 (31%)
Age at first wheeze (months)		12·4 (9·8)	13·5 (10·5)	12·8 (10·1)	12·4 (10·4)	13·6 (11·5)	12·9 (10·8)
Children with episodic viral wheeze		296 (71%)	181 (72%)	477 (71%)	295 (69%)	191 (76%)	486 (72%)
Children with multitrigger wheeze		120 (29%)	72 (28%)	192 (29%)	131 (31%)	60 (24%)	191 (28%)
Interval between onset of URTI and wheezing (h)‡		31·6 (27·4)	28·8 (25·2)	30·5 (26·6)	27·3 (23·4)	28·2 (26·0)	27·7 (24·4)
Children with more than one hospital admission for wheeze in the past year		363 (87%)	216 (85%)	579 (87%)	351 (82%)	203 (81%)	554 (82%)
Courses of oral corticosteroids in past year		2·0 (1·9)	1·8 (1·8)	1·9 (1·8)	1·9 (1·9)	1·8 (2·0)	1·9 (2·0)
USMA in previous year		5·5 (4·3)	5·4 (4·1)	5·4 (4·2)	5·7 (5·3)	5·6 (4·6)	5·6 (5·1)
Continuous inhaled corticosteroids		118 (28%)	66 (26%)	184 (28%)	144 (34%)	69 (27%)	213 (31%)

Data are mean (SD) or n (%), unless otherwise indicated. USMA=unscheduled medial attendance for wheeze. URTI=upper-respiratory-tract infection.

* A question to parents from the International Study of Asthma and Allergies in Childhood questionnaire was used to identify symptoms suggestive of eczema.

† Eczema from birth was based on parental report to recruiting investigator at enrolment.

‡ Based on parental report of the usual interval between URTI and onset of wheezing.

Overall, we recorded 1310 unscheduled medical attendances for wheeze episodes in the montelukast group and 1480 such attendances in the placebo group. There was no difference in mean medical attendances between the montelukast and placebo groups (table 2). These conclusions remained the same when the analysis was repeated in the per-protocol population. Compared with placebo, children in the 5/5 *ALOX5* promoter stratum had reductions in unscheduled medical attendances for wheeze episodes (table 2). By contrast, there was no difference in medical attendances between children in the montelukast and placebo groups in the 5/x+x/x stratum (table 2).

**Table 2:** Treatment response in the primary analysis, and by 5/5 and 5/x+x/x strata

	**Montelukast group (n=652)**	**Placebo group (n=656)**	**Adjusted incidence rate ratio (95% CI)**	**p value**	**p_interacttion_**
**Primary analysis**					
USMA episodes	2·0 (2·6)	2·3 (2·7)	0·88 (0·77–1·01)	0·06	..
**Subgroup analysis**					
USMA in 5/5 stratum	2·0 (2·7)	2·4 (3·0)	0·80 (0·68–0·95)	0·01	..
USMA in 5/x+x/x stratum	2·0 (2·5)	2·0 (2·3)	1·03 (0·83–1·29)	0·79	0·08

Data are mean (SD), unless otherwise indicated. We obtained primary outcome data from the phone call that took place every 2 months. USMA=unscheduled medial attendance for wheeze.

No difference was recorded between the montelukast and placebo groups for the number of children who had at least one unscheduled medical attendance for wheeze episodes, the number of wheeze episodes, or the duration of wheeze episodes (table 3). There was also no difference between treatment groups for time to first unscheduled medical attendance (table 3). Time to first hospital admission was increased in the montelukast group (p=0·04; appendix). There was no difference between the montelukast and placebo groups for attendances to accident and emergency (appendix). Mean number of courses of rescue oral corticosteroids were lower in children given montelukast than in those given placebo (table 3), but there was no difference in the proportion of children receiving at least one course of rescue oral corticosteroids (appendix). In the montelukast group, study drugs were reported to be effective by 323 (56%) of 579 parents at the 12-month timepoint; 58 (10%) parents were unsure, and 69 (12%) did not respond. In the placebo group, study drugs were reported to be effective by 299 (52%) of 575 parents; 58 (10%) parents were unsure, and 78 (14%) did not respond.

**Table 3:** Secondary outcomes

	**Montelukast group (n=652)**	**Placebo group (n=656)**	**Point estimate (95% CI)**	**p value**
Children with one or more USMA	426 (65%)	456 (70%)	OR 0·83 (0·66–1·04)	0·10
Time to first USMA (days)*	147 (50–365)	130 (38–)†	HR 0·89 (0·78–1·02)	0·09
Need for rescue oral corticosteroids (courses per child)‡	0·26 (0·7)	0·33 (0·9)	IRR 0·75 (0·58–0·98)	0·03
Wheeze episodes‡	2·7 (2·9)	2·6 (3·0)	IRR 1·02 (0·91–1·16)	0·68
Duration of wheeze episodes (days)	5·2 (4·0)	5·4 (3·8)	IRR 0·97 (0·89–1·06)	0·53
Duration of hospital admission (days per admission)	1·8 (1·3)	1·7 (1·1)	IRR 1·05 (0·94–1·18)	0·40
Symptomatic days per wheeze episode	4·9 (3·5)	4·8 (3·8)	IRR 0·96 (0·88–1·05)	0·36

Data are n (%), median (IQR), or mean (SD), unless otherwise indicated. USMA=unscheduled medical attendance for wheeze episodes. OR=odds ratio. HR=hazard ratio. IRR=incidence rate ratio.

* Seven participants were missing dates for USMA and seven participants had their first medical attendance on the day of randomisation and were hence excluded.

† The 75th percentile could not be calculated for this IQR because more than 25% of children never had a USMA.

‡ Analysis included all children. 446 children had no diary data and these participants were considered to have no wheeze and cold episodes. When the analysis was repeated with these patients treated as missing, there was no difference in the IRR between treatment and placebo.

There was no significant interaction for pattern of wheeze at baseline (multitrigger *vs* episodic wheeze), use of regular inhaled corticosteroids, or a different grouping of *ALOX5* promoter genotype 5/5+5/x and x/x (appendix).

Of the 940 adverse events reported in the study, 657 (70%) were classified as definitely not related to study drug, 179 (19%) as probably not related, 93 (10%) as possibly related, 11 (1%) as probably related, and no adverse event was definitely related (appendix). We recorded one serious adverse event, which was a skin reaction in a child allocated to placebo (appendix). The distribution of adverse events was similar between groups (table 4).

**Table 4:** Non-serious adverse events

		**Montelukast (n=669)**	**Placebo (n=677)**
Number of events*		397	543
Participants with events		197 (29%)	235 (35%)
Intensity			
	Mild	314 (79%)	426 (78%)
	Moderate	77 (19%)	108 (20%)
	Severe	6 (2%)	9 (2%)
Minor injury		27 (7%)	22 (4%)
Gastrointestinal		86 (22%)	122 (22%)
Upper-respiratory-tract infection		73 (18%)	103 (19%)
CNS		25 (6%)	46 (8%)
Minor infection		87 (22%)	107 (20%)
Allergy		16 (4%)	20 (4%)
Cutaneous		32 (8%)	54 (10%)
Respiratory		34 (9%)	54 (10%)
Haematological		5 (1%)	7 (1%)
Genitourinary		10 (3%)	6 (1%)
Major injury		2 (1%)	1 (<1%)
Musculoskeletal		0	1 (<1%)

Data are n (%), unless otherwise indicated. See appendix for full details of adverse events.

* No adverse events were definitely treatment-related

**Figure 2:**
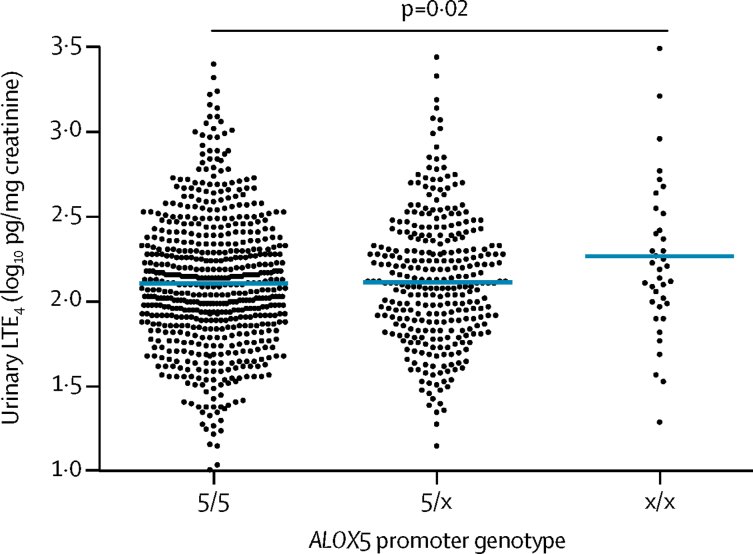
Dot plot of urinary LTE_4_ by variable numbers of copies of the Sp1-binding motif (either 5/5, 5/x, or x/x, in which x does not equal 5) in the *ALOX5* promoter region 11 datapoints were outside the axis and are not shown for convenience. Horizontal bars within plots represent mean. LTE_4_=leukotriene E_4_. *ALOX5*=arachidonate 5-lipoxygenase.

Urine was obtained from 975 asymptomatic children at recruitment. We excluded children with concentrations of urinary creatinine of less than 0·1 mg/mL (n=26), resulting in analysis of 597 (63%) children with the 5/5 genotype, 312 (33%) with the 5/x genotype, and 40 (4%) with the x/x genotype. Urinary leukotriene E4 (log_10_ transformed) was higher in children with the x/x genotype than in those with the 5/5 genotype (figure 2). There was no significant difference in urinary leukotriene E4 between the 5/5 and 5/x genotypes, or the 5/5 and 5/x+x/x genotypes (data not shown).

## Discussion

Our findings show that intermittent montelukast treatment, although not associated with side-effects, did not reduce unscheduled medical attendances for wheeze episodes in children younger than 5 years. These results are in line with those of Bacharier and colleagues,^8^ who reported that intermittent montelukast in young children with wheeze does not increase the proportion of episode-free days or decrease the proportion of children who need urgent medical care, and with those of Valovirta and colleagues^9^ who noted that intermittent montelukast does not reduce the number of wheeze episodes culminating in need for unscheduled medical care or rescue oral corticosteroids. Use of oral steroid rescue therapy in our study was much lower than unscheduled medical attendances for wheeze episodes. We postulate that this finding shows a change in UK prescribing practice in view of studies reporting oral steroids to be ineffective in acute wheeze in young children.^4^, ^16^ We recorded a reduction in use of oral corticosteroid in children given montelukast, but in the context of present UK prescribing practice, the clinical significance of a change in this indirect marker of wheeze severity is unclear. Our results differ to those from Robertson and colleagues^7^ who, in a subgroup analysis, showed that intermittent montelukast is effective in reducing unscheduled use of health-care resources in children aged 2–5 years. To resolve these contradictory findings, we did a meta-analysis of trials of intermittent montelukast for unscheduled medical attendances for wheeze episodes (appendix). Findings of this meta-analysis showed no benefit of a 12 month period of intermittent montelukast therapy on unscheduled medical attendances for wheeze (appendix). This outcome suggests that intermittent montelukast is not an effective treatment strategy for treatment of young children with a history of two or more episodes of wheeze (panel).

Panel **Research in Context**

**Systematic review**

We did a search between June 30, and July 10, 2014, using the research strategy reported by Ducharme and colleagues.^20^ We searched Embase, Scopus, Medline, and the Cochrane Airways Group trials register for additional studies between Jan 1, and July 30, 2014, with search terms “wheez* or asthm*”, “preschool* or preschool**”, “randomised or randomized or randomly or trial”, “leukotriene* or anti-leukotriene or antileukotriene or montelukast”. We also included “viralwheeze or viral-wheeze”, “young children and infant”, “intermittent, pre-emptive, and preemptive”. Our search retrieved no additional trials to those previously identified.^7^, ^8^, ^9^

**Interpretation**

Whether intermittent treatment with montelukast is effective for treatment of wheeze in children aged 10 months to 5 years is unclear: one randomised trial^7^ showed that intermittent montelukast is effective for wheeze in that population, whereas two other trials^8^, ^9^ reported no benefit. We therefore sought to establish the efficacy of intermittent montelukast in young children with wheeze. Because young children with wheeze exhibit marked heterogeneity in response to montelukast, and in adults, copy numbers of the GGGCGG Sp1 binding motif in the arachidonate 5-lipoxygenase (*ALOX5*) gene promoter (either 5/5, 5/x, or x/x, in which x does not equal 5) are associated with heterogeneity in montelukast response,^14^, ^15^ we stratified the trial by 5/5 and 5/x+x/x genotypes. Our findings show that intermittent montelukast is no better than placebo for reducing the need for unscheduled medical attention in young children with a history of clinically severe wheeze. Evidence suggested that children with the 5/5 genotype might be responsive to intermittent montelukast treatment. For clinicians, these data suggest that intermittent montelukast should not be routinely used to treat wheeze in young children. Further data from stratified trials are needed before treatment is targeted to a responsive subgroup.

In the present study, the 95% CI of the IRR for unscheduled medical attendances for wheeze excluded a 33% reduction in such attendances. However, the fewer unscheduled medical attendances in the montelukast group, albeit non-significant, suggests heterogeneity of treatment response—a characteristic of previous studies in young children with wheeze. For example, response to continuous inhaled corticosteroids is most favourable in the subgroup of white males with an unscheduled medical attendance for wheeze in the previous 12 months and aeroallergen sensitisation.^21^ Furthermore, in Bacharier and colleagues' study,^8^ intermittent montelukast, despite having no overall benefit, reduced the area under the curve for wheezing score in children with a positive asthma predictive index, defined as four or more wheezing episodes with at least one diagnosed by a doctor, and one or more major criteria of parental asthma, doctor-diagnosed dermatitis, allergic sensitisation to one or more aeroallergen, or at least two minor criteria of allergic sensitisation to milk, egg, or peanuts; wheeze unrelated to colds; and blood eosinophils greater than 4%.^22^ We did not stratify by asthma predictive index because Meyer and colleagues^22^ reported that no clinical variable predicts response to continuous montelukast in wheeze in young children, and blood sampling, in our experience, greatly reduces the willingness of parents to enter their infants into a therapeutic trial. Furthermore, use of parental-reported diagnosis for disorders such as eczema overestimates physician-diagnosed disease.^23^ As such, we cannot exclude montelukast responsiveness in children with a positive asthma predictive index. However, our prespecified subgroup analyses showed that neither the pattern of wheeze nor use of inhaled corticosteroids was associated with montelukast response, although our study was not powered for these interactions.

In adults with asthma, heterogeneity in response to montelukast is associated with a polymorphism in the *ALOX5* promoter.^14^, ^15^ In line with these studies in adults, we recorded a 20% reduction in unscheduled medical attendances for wheeze in children in the montelukast group with the 5/5 *ALOX5* promoter genotype, and no effect of intermittent montelukast in those with the 5/x+x/x genotype. The montelukast-responsive genotype (5/5) in the present study is, however, different from our a-priori hypothesis, as suggested by the 5/x+x/x grouping from Lima and colleagues' study.^14^ But other studies in adults report montelukast responsiveness of the 5/5 genotype. For example, Telleria and colleagues^15^ reported decreased asthma exacerbations and improved lung function in adults with the 5/5 genotype who were given montelukast, and Drazen and colleagues^24^ showed that ABT-761 (a 5-lipoxygenase inhibitor) improved lung function in adults with the 5/5 genotype, but not in those with the x/x genotype. We sought support for a differential response to montelukast between genotypes by measurement of urinary leukotriene E_4_.^25^ In the only study in children to date, Mougey and colleagues^19^ measured urinary leukotriene E_4_ and identified *ALOX5* polymorphism status in 270 6–17-year-old children with poorly controlled asthma enrolled into a 6 month (negative) trial of acid-reflux treatment. Children with the x/x genotype (73% of whom were receiving montelukast) had significantly higher concentrations of urinary leukotriene E_4_, worse forced expiratory volume in 1 s, and a trend for poorer asthma control than those with the 5/5+5/x genotypes.^19^ Similarly, we recorded increased urinary leukotriene E_4_ in children with the x/x genotype compared with those with the 5/5 or 5/5+5/x genotypes. These data provide support for a differential response to montelukast between 5/5 and x/x genotypes; however, they do not explain a differential response between the 5/x and 5/5 genotypes. We postulate that differences in production of cysteinyl leukotriene between 5/x and 5/5 genotypes might be shown during children's wheeze episodes when cysteinyl leukotriene production is increased.^26^

These data do not support the routine use of intermittent montelukast for wheeze in children aged 10 months to 5 years. Further stratified trials should be done to confirm the presence of a responsive subgroup.
