# Supplementary material for: Intermittent montelukast in children aged 10 months to 5 years with wheeze (WAIT trial): a multicentre, randomised, placebo-controlled trial
Source: Lancet Respir Med. 2014 Oct;2(10):796–803. doi: 10.1016/S2213-2600(14)70186-9 (PMC4189104; doi:10.1016/S2213-2600(14)70186-9)
Supplement: Supplementary appendix [file mmc1.pdf]

THE LANCET Respiratory Medicine

Supplementary appendix

This appendix formed part of the original submission and has been peer reviewed. We post it as supplied by the authors.

Supplement to: Nwokoro C, Pandya H, Turner S, et al. Intermittent montelukast in children aged 10 months to 5 years with wheeze (WAIT trial): a multicentre, randomised, placebo-controlled trial. *Lancet Respir Med* 2014; published online Sept 9. http://dx.doi.org/10.1016/S2213-2600(14)70186-9.

Supplementary Appendix for;

Intermittent montelukast in children 10 months - 5years with wheeze (WAIT trial): a multicentre, randomised, placebo-controlled trial

Chinedu Nwokoro MBBChir [1], Hitesh Pandya MD [3], Stephen Turner MD [4], Sandra Eldridge PhD[1], Christopher J Griffiths MD [1], Tom Vulliamy PhD [1], David Price MRCGP [4], Marek Sanak PhD [5], John W Holloway PhD [6], Rossa Brugha BMBCh [1], Lee Koh BSc [1], Iain Dickson MRes [1], Clare Rutterford MSc [2], Jonathan Grigg MD [1]

1. Asthma UK Centre for Applied Research, Queen Mary University of London, London, UK

2. Centre for Primary Care and Public Health, Blizard Institute, Queen Mary University of London, London, UK

3. Department of Infection, Immunity and Inflammation, University of Leicester, Leicester, UK

4. University of Aberdeen, Aberdeen, UK.

5. Department of Medicine, Jagiellonian University Medical School, Krakow, Poland

6. Human Development and Health, University of Southampton, Southampton General Hospital, UK

Methods

Genotyping

Samples were collected from children using the OG-250 Oragene DNA Self-Collection Kit (DNA Genotek, Ottawa, Canada) in combination with the saliva collection kit for young children. Genomic DNA was extracted from these samples according to the manufacturer's instructions. The simple sequence length polymorphism in the promoter of ALOX5 (rs59439148) was genotyped as previously described [1]. FAM-labelled amplicons were sized on a 3130xl Genetic Analyser (Life Technologies, Paisley UK). For the analysis, 20ng of genomic DNA was added to a reaction mix containing 1x PCR buffer and 0.5 U of AmpliTaq Gold DNA polymerase (Applied Biosystems) with 2.5mM $MgCl_2$, 5% DMSO, 0.2µM dNTPs containing a 3:1 ratio of dGTP to 7-deaza-dGTP and 0.2µM of each primer in a final volume of 20µl. The primer sequences were 5'FAM-AGGAACAGACACCTCGCTGAGGAGAG-3' and 5'GAGCAGCGAGCGCCGGGAGCCTCGGC3'. Cycling conditions were 95°C for 6 min followed by 35 cycles of 95°C for 15s, 62°C for 23s and 72°C for 30s followed by a final extension at 72^{0}C for 5 minutes. Products of the reaction were diluted 1:5 in water and 1µl of this dilution was added to 9µl Hi-Di™ formamide (Life Technologies, Paisley, UK) + 0.3µl ROX500 size standard (Applied Biosystems) and analysed by capillary electrophoresis on a 3130xl Genetic Analyser (Applied Biosystems). Fragments of 256 to 292bp were obtained depending on the copy number (2 to 8) of the repeat sequence and were visualized using GeneMapper v4.0 or Peak Scanner v1.0 software. Genotypes were called manually from duplicate amplifications. Samples with known genotypes, which had been verified by DNA sequence analysis, were included in each run. Alleles were called according to the

number of simple repeats. Samples with the most common genotype (homozygous 5/5) were allocated to one stratum. Samples with any other genotype (either 5/x or x/x, where x is any allele other than 5 were allocated to the [5/x+x/x] stratum.

Urinary leukotriene E_4

Urine samples were obtained from children immediately following sputum induction and stored on ice until transfer to storage at -80 ^{o}C within 1 h. Analysis was by gas chromatography-mass spectrometry using negative ion chemical ionisation (GC—NICI-MS). To each urine sample (0.5 mL), chemically identical deuterated internal standards (1ng, Cayman Chemical Company, AnnArbor, MI) were added, acidified with acetic acid (pH 4.5), then extracted twice with methyl tertbutyl-ether and dried under nitrogen. Methanol dissolved aliquots (10µL, in methanol) were injected onto a reverse phase column (Zorbax Eclipse XDB C-18, Agilent Technologies, Inc. Santa Clara, CA, USA), stabilised thermally at 37 ^{o}C and a gradient consisting of two mobile phases: A acetonitrile/water/acetic acid (20/80/0.0001) and B acetonitrile/iso-propanol/acetic acid (55/45/0.0001, v/v) was used to elute LTE4 with the flow rate 0.11 mL/min using HPLC equipped with an autosampler (Shimadzu Sil-2-AC, Shimadzu Scientific Instruments, Inc. Columbia, MD, USA). The mobile phase binary linear gradient was 1 min 8% B, 9.5 min 8 to 95% B, 0.5 min 95% B, 0.5 min 95–100% B, 2 min 100% B. Leukotriene E_4 was measured using multiple reaction monitoring mode (MRM) tandem mass spectrometry (Qtrap 4000, Applied Biosystems, Foster City, CA, USA) equipped with an electrospray ion source. Urine sample extract was prepared by a 2 step derivatisation to pentafluorobenzyl and trimethylsilyl esters which modified carboxyl and hydroxyl groups of the compound,

and were purified by a thin-layer chromatography. A gas chromatography negative–ion chemical ionisation mass–spectrometry was used for quantification (model Engine 5989B series II Helwett Packard, Palo Alto, CA). All solvents were HPLC grade and purchased from Mallincrodt Baker, Inc. Phillipsburg, NJ, USA), while other chemicals were from Sigma–Aldrich Co. St. Louis, MO, USA).

Combined analysis

In order to compare the primary outcome of this study to published reports, we undertook a review of previously published data based on the non-systematic review approach described by Ducharme and colleagues [2] for the period January 1980 to January 2014. Two authors (JG and RB) searched EMBASE, SCOPUS, MEDLINE and the Cochrane Airways Group trials register for details of trials published after the review by Ducharme and colleagues [2]. We sought further trials from 1st January 2014 to 1st August 2014 with a placebo-controlled design that assessed the efficacy of intermittent montelukast with respect to unscheduled medical attendances for wheezing in pre-school children over a 12-month follow-up. We identified no additional studies were identified. The three studies reported by Ducharme and colleagues [2] were therefore selected for the combined analysis; Robertson and colleagues [3], Valovirta and colleagues [4], and Bacharier and colleagues [5].

The published reports were reviewed for details on pre-specified outcomes. The study by Robertson and colleagues [3] reported "unscheduled health care resource utilisations (HRU) in the group of children who received at least one episode of treatment as a

primary outcome", in children between two to 14 years of age. Raw data from this study were provided by personal communication (by author DP), from which we calculated the mean and standard deviation (SD) for the number of episodes requiring an unscheduled medical attendance for each child within the subgroup of children aged two to five years. The study by Bacharier and colleagues [5] enrolled children aged one to six years, with number of "unscheduled visits to primary care offices, urgent care or emergency departments and hospitalisations for acute wheezing episodes" as a secondary outcome. The mean and standard deviation for these episodes was published in their report, additionally the number of children with one or more events for this outcome was confirmed by personal correspondence (Dr Bacharier). Original data were not received from the study of Valovirta and colleagues [4] which included children aged six months to five years, and reported a secondary outcome for "adjusted rate of asthma attacks", with an attack defined within the statement; "the start of an asthma attack was the first day the patient's symptoms required HRU". Overall, the 3 studies were comparable in terms of study design (placebo-controlled trial), randomisation, concealment of allocation, inclusion criteria and duration of follow-up (12 months) to those in this report (Supplementary Table 7).

A meta-analysis was performed using the inverse variance fixed effect method to calculate the summary weighted difference between the means with 95% confidence intervals. The analysis was performed using RevMan version 5.3 [6]. We compared the mean (SD) for number of episodes requiring an unscheduled medical attention per child in each study, comparing experimental (intermittent montelukast) and control (placebo) groups, using a fixed effects model for mean difference as per the review by

Ducharme and colleagues [2]. The overall analysis of 2783 children shows no overall benefit for intermittent montelukast therapy in reducing need for unscheduled medical attention for wheeze (weighted mean difference = -0.10, 95% CI -0.26 to -0.06, test for overall effect p=0.21, Fig. S2, Appendix).

Table S1. Enrolment questionnaire

T -2 ASSESSMENT & RANDOMISATION CRF (Copy 1 – Trial Manager, Copy 2 – Local Site File)

Serial number: I__I__I__I__I Site: I__I__I (e.g LO, AB, LE)

Researcher Initials:I__I__I Date of THIS Visit: I__I__/__I__/__I__I

Weight:	I__I__I.I__Ikg	Height:	I__I__I__I.I__Icm	DOB:	I__I__/__I__/__I__I	Sex	M ☐ F ☐

Risk factors

**Birth, Atopy and Family History**	**Yes**	**No**
Preterm Birth < 37wk gestation	☐	☐
Birth weight < 2500g	☐	☐
Allergy:		
Food	☐	☐
Drug	☐	☐
Itchy rash for > 6 months, ever	☐	☐
Eczema, ever	☐	☐
Tobacco Exposure:		
In utero	☐	☐
In household[+] (+any household smoking contact)	☐	☐
Daycare attendance	☐	☐
Immunisation Status:		
Pneumococcus	☐	☐
Influenza	☐	☐
History of Asthma		
Mother:	☐	☐
Father:	☐	☐

**Pre-study Illness and Therapy**		
Age at 1st wheeze episode	I__Iy I__I__Im	
	**Yes**	**No**
Wheezes only with viral URTI (episodic)	☐	☐
Wheezes at other times (multitrigger)	☐	☐
Interval between onset of URTI and wheezing:	I__I__I hr	
Admitted to hospital for wheeze:		
In last year?	☐	☐
Ever?	☐	☐
No of courses of systemic steroids in last year	I__I__I	
No of unscheduled medical attendances for wheeze in last year?	I__I__I	
Preventer therapy:		
None	☐	☐
Antileukotriene agents	☐	☐
Maintenance Inhaled Steroids	☐	☐
Episodic inhaled Steroids	☐	☐

Ethnicity

**Asian or Asian British**	**Mixed**	**Black or Black British**	**White**	**Other Ethnic Group**
☐ Bangladeshi	☐ White & Asian	☐ African	☐ British	☐ Chinese
☐ Indian	☐ White & Black African	☐ Caribbean	☐ Irish	☐ Any other ethnic group
☐ Pakistani	☐ White & Black Caribbean	☐ Any other Black background	☐ White other	☐ I do not wish to disclose my ethnic origin
☐ Any other Asian background	☐ Mixed other			

Saliva sample collected:	Yes ☐	No ☐	Date collected:	I__I__/__I__/__I__I
Saliva sample posted to laboratory:	Yes ☐	No ☐	Date sent:	I__I__/__I__/__I__I
Urine sample collected:	Yes ☐	No ☐	Date collected:	I__I__/__I__/__I__I

STUDY VISIT CONDUCTED BY:

Researcher Signature:_______________ Print Name:________________________ I__I__/__I__/__I__I

I have reviewed all data in this CRF and verify that the contents are consistent with observations and source records.

PI Signature_______________________ Print Name:________________________ I__I__/__I__/__I__I

Table S2. Parent diary card for the first day of starting trial medication for a wheeze episode

DAY 1 OF MEDICINE

SUN MON TUES WED THUR FRI SAT

DATE: I__I__/__I__/20__I__I TIME: __I__:__I__

The questions below refer to the past 24 hours.

Please answer as well as you can remember

Please answer the questions by ticking (√) Yes or No →	Y	N
Did your child wheeze in the last 24 hours?		
Did your child have a cold in the last 24 hours?		
Did you give your child the TRIAL medicine TODAY?		
Did your child vomit the medicine TODAY?		
Did your child miss school or nursery TODAY?		
Did **ANYONE** stay home to look after your child TODAY?		
Did your child see a doctor or nurse TODAY?		
Did you give your child the blue inhaler in the last 24 hrs		
If yes? How many times did you give it to them in the last 24 hours?		
On average, how many puffs did you give them each time?		

Parent Initials I__I__I WAIT DIARY CARD, v3 280610 **2**

Table S3. The intention to treat and per protocol populations

	Montelukast		Placebo	
**ITT Population**	**n=669**	**(50%)**	**n=677**	**(50%)**
**Timing of last contact**				
**T0 (no data)**	17	(3%)	21	(3%)
**Withdrew before T1**	16/17		16/21	
**T1 (month 2)**	21	(3%)	20	(3%)
**T2 (month 4)**	15	(2%)	12	(2%)
**T3 (month 6)**	12	(2%)	19	(3%)
**T4 (month 8)**	13	(2%)	15	(2%)
**T5 (month 10)**	12	(2%)	15	(2%)
**T6 (month 12)**	579	(87%)	575	(85%)
**Per protocol population**	647		650	

ITT; intention to treat. T; phone call to parents

Table S4. Distribution of ALOX5 promoter polymorphism genotype by parent-reported ethnicity

Genotype	White n (%)	Black n (%)	Asian n (%)	Bangladeshi n (%)	Mix n (%)	Other n (%)	All n (%)
**3/3**	0 (0.00)	4 (10.81)	0 (0.00)	0 (0.00)	0 (0.00)	0 (0.00)	4 (0.29)
**3/4**	1 (0.10)	2 (5.41)	0 (0.00)	1 (0.75)	2 (2.78)	1 (2.70)	7 (0.51)
**3/5**	4 (0.39)	6 (16.22)	0 (0.00)	0 (0.00)	10 (13.89)	4 (10.81)	24 (1.76)
**3/6**	0 (0.00)	2 (5.41)	0 (0.00)	0 (0.00)	0 (0.00)	0 (0.00)	2 (0.15)
**3/7**	0 (0.00)	0 (0.00)	0 (0.00)	0 (0.00)	1 (1.39)	1 (2.70)	2 (0.15)
**4/4**	18 (1.75)	0 (0.00)	8 (13.34)	5 (3.73)	2 (2.78)	1 (2.70)	34 (2.49)
**4/5**	285 (27.78)	10 (27.03)	18 (30)	33 (24.63)	11 (15.28)	7 (18.92)	364 (26.65)
**4/6**	6 (0.58)	0 (0.00)	1 (1.67)	2 (1.49)	0 (0.00)	1 (2.70)	10 (0.73)
**5/5**	677 (65.98)	9 (24.32)	27 (45)	83 (61.94)	43 (59.72)	19 (51.35)	858 (62.81)
**5/6**	30 (2.92)	4 (10.81)	5 (8.33)	10 (7.46)	3 (4.17)	2 (5.41)	54 (3.95)
**6/6**	0 (0.00)	0 (0.00)	1 (1.67)	0 (0.00)	0 (0.00)	0 (0.00)	1 (0.07)
**2/4**	1	0	0	0	0	0	1

	(0.10)	(0.00)	(0.00)	(0.00)	(0.00)	(0.00)	(0.07)
**5/8**	1	0	0	0	0	0	1
	(0.10)	(0.00)	(0.00)	(0.00)	(0.00)	(0.00)	(0.07)
**5/7**	2	0	0	0	0	0	2
	(0.19)	(0.00)	(0.00)	(0.00)	(0.00)	(0.00)	(0.15)
**2/5**	1	0	0	0	0	0	1
	(0.10)	(0.00)	(0.00)	(0.00)	(0.00)	(0.00)	(0.07)
**3/8**	0	0	0	0	0	1	1
	(0.00)	(0.00)	(0.00)	(0.00)	(0.00)	(2.70)	(0.07)
**Total**	1026	37	60	134	72	37	1366*
	(100)	(100)	(100)	(100)	(100)	(100)	(100)

**Stratum**	**White**	**Black**	**Asian**	**Bangladeshi**	**Mix**	**Other**	**All**
**5/5**	677	9	27	83	43	19	858
	(65.98)	(24.32)	(45.00)	(61.94)	(59.72)	(51.35)	(62.81)
**5/X**	323	20	23	43	24	13	446
	(31.48)	(54.05)	(38.33)	(32.09)	(33.33)	(35.14)	(32.65)
**X/X**	26	8	10	8	5	5	62
	(0.19)	(21.62)	(16.67)	(5.97)	(6.94)	(13.51)	(4.54)
**Total**	1026	37	60	134	72	37	1366*
	(100)	(100)	(100)	(100)	(100)	(100)	(100)

*Of the 1366 children genotyped, 8 were withdrawn prior to randomisation

Table S5: Time to first unscheduled medical attendance for wheeze by site of attendance

ITT population<br>Time (in days) to first:	Montelukast<br>N=652<br>median days (IQR)	Placebo<br>N=656<br>median days (IQR)	Hazard Ratio<br>(95% CI)	p-value
**Any (N=1294)***	147 (50,365)	130 (38,.)	0.89 (0.78, 1.02)	0.10
**Hospital admission (N=1305)**	. (202, .)	. (144, .)	0.82 (0.68, 0.99)	0.04
**Accident and emergency department only (N=1308)**	. (.,.)	. (.,.)	0.89 (0.53, 1.52)	0.68
**Unscheduled GP visit (N=1297)**	257 (64,365)	240 (68, 365)	0.94 (0.81, 1.09)	0.41

*7 participants are missing dates for the medical attendances and 7 participants had their first medical attendance on the day of randomisation and are hence excluded.

Data are analysed using a Cox regression model with fixed effects for stratification factor and treatment group. (.) is where the value has not been observed since there was less than the required proportion of children who experienced an event within the follow up period.

Table S6: Medication usage

Intention to treat population	Placebo N=677	Montelukast N=669	IRR or OR (95% CI)	p-value
**Proportion of children receiving oral corticosteroids, N (%)***	233 (34%)	227 (34%)	OR; 0.98 (0.78 to 1.23)	0.86
**Number of sachets of trial medication used per diary card, mean (SD)****	9.4 (1.5)	9.4 (1.6)	IRR; 0.98 (0.96 to 1.00)	0.09

*Data are analysed using logistic regression with fixed effects for stratification factor and treatment group

**Data are analysed using Poisson regression with fixed effects for stratification factor and treatment group and a random effect for child to account for diary card aas the unit of analysis - with follow up time fitted as the exposure.

OR; odds ratio, IRR incidence rate ratio.

Table S7. Unscheduled medical attendance for wheeze episodes in pre-specified subgroups

	Montelukast Group		Placebo Group		
	**Mean**	**(SD)**	**Mean**	**(SD)**	p_{int}
**Alternative genotype grouping**					
**[5/5 + 5/x]**	2.02	(2.63)	2.27	(2.80)	0.93
**[x/x]**	1.68	(1.84)	1.91	(1.99)	
**ICS use at baseline**					
**ICS at baseline**	2.04	(3.02)	1.99	(2.34)	0.09
**No ICS at baseline**	1.99	(2.24)	2.45	(3.00)	
**Wheeze pattern at baseline**					
**Multi-trigger wheeze**	2.05	(2.96)	2.03	(2.45)	0.19
**Episodic wheeze**	1.99	(2.44)	2.34	(2.88)	

ICS; inhaled corticosteroids, $p_{int.}$; P-value for interaction, Multi-trigger wheeze; wheeze with and without viral colds, Episodic wheeze; wheeze only with colds

Table S8. **Relationship of adverse events to study medication.**

	Montelukast (N=669) N (%)	Placebo (N=677) N (%)	Total (N=1346) N (%)
**Total number of events: definitely not related**	**281**	**376**	**657**
Minor injury	27 (10%)	22 (6%)	49 (7%)
Gastrointestinal	40 (14%)	62 (16%)	102 (16%)
Upper respiratory tract infection	63 (22%)	88 (23%)	151 (23%)
Central nervous system	8 (3%)	10 (3%)	18 (3%)
Minor infection	76 (27%)	91 (24%)	167 (25%)
Allergy	13 (5%)	16 (4%)	29 (4%)
Cutaneous	18 (6%)	32 (9%)	50 (8%)
Respiratory	25 (9%)	47 (13%)	72 (11%)
Haematological	2 (1%)	2 (1%)	4 (1%)
Genitourinary	7 (2%)	4 (1%)	11 (2%)
Major injury	2	1	3

	(1%)	(<1%)	(<1%)
Musculoskeletal	0	1	1
		(<1%)	(<1%)
**Total number of events: probably not related**	**80**	**99**	**179**
Minor injury	0	0	0
Gastrointestinal	26	33	59
	(33%)	(33%)	(33%)
Upper respiratory tract infection	10	15	25
	(13%)	(15%)	(14%)
Central nervous system	5	8	13
	(6%)	(8%)	(7%)
Minor infection	11	16	27
	(14%)	(16%)	(15%)
Allergy	3	4	7
	(4%)	(4%)	(4%)
Cutaneous	10	13	23
	(13%)	(13%)	(13%)
Respiratory	9	7	16
	(11%)	(7%)	(9%)
Haematological	3	1	4
	(4%)	(1%)	(2%)
Genitourinary	3	2	5
	(4%)	(2%)	(3%)
Major injury	0	0	0
Musculoskeletal	0	0	0
**Total number of events: possibly related**	**33**	**60**	**93**

Minor injury	0	0	0
Gastrointestinal	19 (58%)	23 (38%)	42 (45%)
Upper respiratory tract infection	0	0	0
Central nervous system	10 (30%)	25 (42%)	35 (38%)
Minor infection	0	0	0
Allergy	0	0	0
Cutaneous	4 (12%)	8 (13%)	12 (13%)
Respiratory	0	0	0
Haematological	0	4 (7%)	4 (4%)
Genitourinary	0	0	0
Major injury	0	0	0
Musculoskeletal	0	0	0
**Total number of events: probably related**	**3**	**8**	**11**
Minor injury	0	0	0
Gastrointestinal	1 (33%)	4 (50%)	5 (45%)
Upper respiratory tract infection	0	0	0
Central nervous system	2 (67%)	3 (38%)	5 (45%)
Minor infection	0	0	0
Allergy	0	0	0
Cutaneous	0	1 (13%)	1 (9%)

Respiratory	0	0	0
Haematological	0	0	0
Genitourinary	0	0	0
Major injury	0	0	0
Musculoskeletal	0	0	0
**Total number of events: definitely related**	**0**	**0**	**0**
Minor injury	0	0	0
Gastrointestinal	0	0	0
Upper respiratory tract infection	0	0	0
Central nervous system	0	0	0
Minor infection	0	0	0
Allergy	0	0	0
Cutaneous	0	0	0
Respiratory	0	0	0
Haematological	0	0	0
Genitourinary	0	0	0
Major injury	0	0	0
Musculoskeletal	0	0	0

Table S9 Studies included in meta-analysis

Study	Inclusion criteria	Design	Follow-up	Data provided	Outcome	Definition of outcome
**Bacharier and colleagues 2008** [5]	- Age 12 to 59 months<br>- 2 or more episodes of wheeze with RTI in past year	3 arm double-blind RCT. 7 days intermittent montelukast 4mg od po vs. budesonide vs. placebo. Parent-initiated therapy.	12 months	Yes – for montelukast and placebo subgroups	Secondary outcome: number of unscheduled visits for acute wheezing episodes	Visit to primary care office, urgent care, ED or hospitalisation
**Robertson and colleagues 2007** [3]	- Age 2 to 14 years<br>- Doctor diagnosed intermittent asthma<br>- Between 3 to 6 exacerbations in past 12 months	2 arm double blind RCT. 7 days (but up to maximum of 20 days if required) montelukast 4mg od po (in 2 to 5 yr subgroup) vs. placebo. Parent-initiated	12 months	Yes – full data set provided. Analysis of 2 to 5 years subgroup performed.	Primary outcome: unscheduled HRU	Unscheduled visit to GP, specialist Paediatrician, ED or admission to hospital specific for asthma

		therapy.				
**Valovirta and colleagues 2011** [4]	- Age 6 months to 5 years<br>- episodes of asthma symptoms in past 12 months; 2 to 4 if under 2 years, 3 to 6 if over 2 years, at least 1 episode in previous 6 months	3 arm double-blind RCT.<br>12 days montelukast vs. daily montelukast vs. placebo.<br>Parent initiated based on symptom calendar.	12 months	No	Secondary outcome: adjusted rate for number of asthma attacks	- Start of an asthma attack defined as the first day the patient's symptoms required an HRU<br>- only one attack was counted per "episode"

ED; emergency department, HRU; health care resource utilisation, RCT; randomised controlled trial, RTI; respiratory tract infection

Figure S1. **Study design and schedule of procedures.**

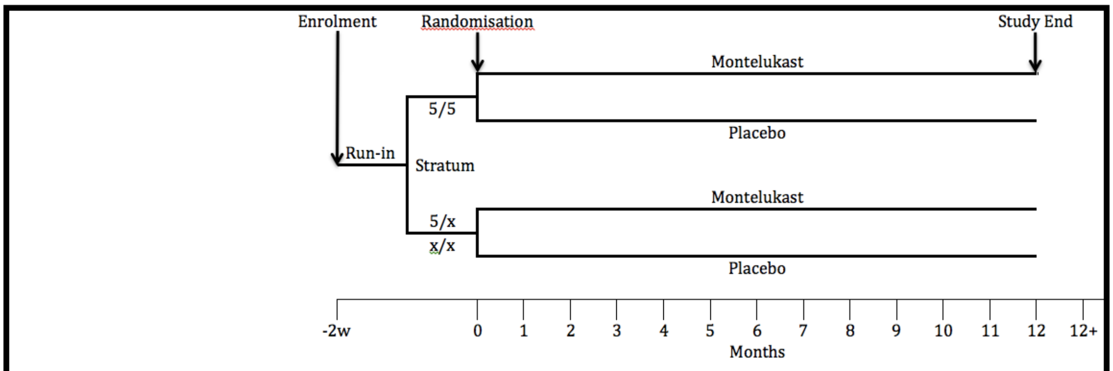

	V1			V2		T1		T2		T3		T4		T5		T6	V3*
Check eligibility	X			X		X		X		X		X		X		X	
Informed consent	X																
Anthropometry and Questionnaire	X																
Review USMA/medications/AEs	X			X		X		X		X		X		X		X	
Saliva sampling	X																
Baseline urine sampling	X																X
Train parents in IMP use				X													
Check inhaler technique				X													
Check ALOX5 status		X															
First IMP dispensing				X													
Repeat IMP dispensing					Variable timing – dependent on expiry/exhaustion of IMP supply												

Children were assigned to receive either montelukast or placebo stratified by 5/5 and [5/x+x/x] arachidonate 5-lipoxygenase gene promoter (ALOX5) promoter genotype. Primary outcome data were taken from the phonecall (T), which occurred every two months. ALOX5; arachidonate 5-lipoxygenase gene promoter, IMP; investigational medicinal product, USMA; need for unscheduled medial attention for wheeze, V; visits to study team

Figure S2. **Meta-analysis of the effect of intermittent montelukast on mean (SD) number of unscheduled medical attendances for wheeze episodes (USMA) in preschool children receiving a 12 month course of intermittent oral montelukast therapy compared with placebo.**

	Experimental			Control				Mean Difference
Study or Subgroup	Mean	SD	Total	Mean	SD	Total	Weight	IV, Fixed, 95% CI
Bacharier 2008	1.5	1.9529	94	1.6	1.7029	47	6.2%	-0.10 [-0.73, 0.53]
Nwokoro 2014	2	2.6	652	2.26	2.7	656	29.7%	-0.26 [-0.55, 0.03]
Robertson 2007	1.71	1.98	79	2.2	2.24	82	5.7%	-0.49 [-1.14, 0.16]
Valovirta 2011	1.23	1.852	588	1.21	1.7241	585	58.4%	0.02 [-0.18, 0.22]
**Total (95% CI)**			**1413**			**1370**	**100.0%**	**-0.10 [-0.26, 0.06]**

Heterogeneity: $Chi^2 = 3.88$, $df = 3$ ($P = 0.27$); $I^2 = 23\%$
Test for overall effect: $Z = 1.25$ ($P = 0.21$)

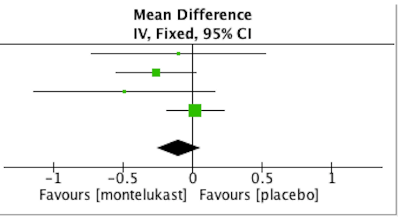

Mean group difference (fixed effects model) is shown. The width of the horizontal line indicates the 95% CI around the point estimate, and the area of the point estimate (square) is a representation of the relative weight of the study in the overall analysis. The pooled summary (diamond) represents the overall result, with the vertical line representing no overall effect (weighted mean difference=0). There is no benefit of intermittent montelukast in an unselected population of children with preschool wheeze (test for overall effect, p=0·21). Nwokoro 2014 refers to the current study

References

1 Sayers I, Barton S, Rorke S, et al. Promoter polymorphism in the 5-lipoxygenase (ALOX5) and 5-lipoxygenase-activating protein (ALOX5AP) genes and asthma susceptibility in a Caucasian population. Clin Exp Allergy 2003; 33:1103-1110

2 Ducharme FM, Tse SM, Chauhan B. Diagnosis, management, and prognosis of preschool wheeze. Lancet 2014; 383:1593-1604

3 Robertson CF, Price D, Henry R, et al. Short-course montelukast for intermittent asthma in children: a randomized controlled trial. Am J Respir Crit Care Med 2007; 175:323-329

4 Valovirta E, Boza ML, Robertson CF, et al. Intermittent or daily montelukast versus placebo for episodic asthma in children. Ann Allergy Asthma Immunol 2011; 106:518-526

5 Bacharier LB, Phillips BR, Zeiger RS, et al. Episodic use of an inhaled corticosteroid or leukotriene receptor antagonist in preschool children with moderate-to-severe intermittent wheezing. J Allergy Clin Immunol 2008; 122:1127-1135 e1128

6 Review Manager (RevMan) Copenhagen: The Nordic Cochrane Centre, The Cochrane Collaboration, 2014

Acknowledgements

Independent members of the trial steering committee; Professor Warren Lenney (Chair) (North Staffordshire NHS Research & Development Institute for Science & Technology in Medicine, Keele University), and Dr Edward Simmonds (University Hospitals Coventry and Warwickshire NHS Trust, Walsgrave General Hospital).

Data monitoring and safety committee; Professor Andrew Bush (Chair) (Imperial College London), Professor Paul Lambert (University of Leicester), Mr Ian Jarrold (British Lung Foundation).

Trial support group; Ms Cassie Brady (data monitoring), Ms Hafiza Khatun (data audit), Ms Suzi Miranbeg (trial co-ordination), Ms Teresa McNally, Ms Donna Nelson (multi-center co-ordination), and Mr Gordon Forbes (statistics).

Local investigators in secondary care centres; Dr Christopher Upton (Norfolk and Norwich University Hospitals NHS Trust), Dr Maria O'Callaghan (Barts Health NHS Trust, Whipps Cross Hospital), Dr S. Murthy Saladi (Countess of Chester NHS Foundation Trust), Dr Catherine Tuffrey (Portsmouth Hospitals NHS Trust), Dr Sheng-Ang Ho (East Cheshire NHS Trust), Dr Robert Ross Russell (Cambridge University Hospitals NHS Trust), Dr Anil Tuladhar (North Tees and Hartlepool NHS Trust), Dr Edwin Osakwe (Oxford Radcliffe Hospitals NHS Trust), Dr Paul McNamara (Alder Hey Children's NHS Trust), Dr James Y Paton (NHS Lothian University Hospitals, Royal Hospital for Sick Children), Dr Mansoor Ahmed (Burton

Hospitals NHS Foundation Trust), Dr John Alexander (University Hospital of North Staffordshire NHS Trust), Dr Deepthi Jyothish (Birmingham Children's Hospital NHS Trust), Dr John Scanlon (Worcestershire Acute NHS Trust), Dr Edward Simmonds (University Hospitals of Coventry NHS Trust), Dr James Crossley (Chesterfield Royal NHS Foundation Trust), Dr Shakeel Rahman (Harrogate and District NHS Foundation Trust), Professor Harish Vyas (Nottingham University Hospitals NHS Trust), Dr Will Carroll (Royal Derby Hospitals NHS Trust), Dr Diarmuid P Kerrin (Barnsley NHS Foundation Trust), Dr Hazel Evans (Southampton University Hospitals NHS Trust), Dr Anna Mathew (Western Sussex NHS Hospitals Trust), Dr Anne Prendiville (Royal Cornwall Hospital Trust), Professor Mark Everard (Sheffield Children's NHS Foundation Trust), Dr Lakshmi Chilukuri (St Helens and Knowsley Teaching Hospitals NHS Trust), Dr Sharryn Gardner (Southport and Ormskirk NHS Trust), Dr Gary Ruiz (King's College Hospital Foundation NHS Trust), Dr Simon Langton Hewer (University Hospitals Bristol NHS Trust), Dr Peter DeHalpert (Royal Berkshire NHS Foundation Trust), Dr Paul Seddon (Brighton and Sussex University Hospitals NHS Trust), Dr Tim Adams (NHS Ayrshire & Arran), Dr David Cremonesini (Hinchingbrooke Health Care NHS Trust), Dr Jonathan Garside (Calderdale and Huddersfield NHS Trust), Dr Anil Shenoy (Bradford Teaching Hospitals NHS Trust), Dr Matthew Babirecki (Airedale NHS Foundation Trust), Dr Anne Ingram (Luton & Dunstable Hospital NHS Trust), Dr John Furness (County Durham and Darlington NHS Trust), Dr David Lacy (Wirral University Teaching Hospital NHS Trust), Dr Mike Linney (Western Sussex Hospitals NHS Trust).

Recruitment centres of study authors. Barts Health NHS Trust, The Royal London Hospital, University Hospitals Leicester NHS Trust, Royal Aberdeen Children's Hospital.

Primary care recruitment centres; Springfield GP-led Health Centre, Lower Clapton Practice, The Lawson Practice, Neaman Practice, Elm Practice, Sandringham Practice, Queensbridge Group Practice, Latimer Health Centre, Statham Grove Surgery. In the Tower Hamlets Primary Care Trust; Strouts Place Medical Centre, Jubilee Street Practice, Wapping Health Centre, East One Health, Barkantine Health Centre, Blithehale Health Centre, Albion Health Centre, Chrisp Street Practice, Bromley-By-Bow Health Centre, XX Place Surgery, St Andrews Health Centre, Mission Practice.
